# Identified *Neptunicella plasticusilytica* sp. nov. and its novel PET-degrading enzyme derived from mangrove plastic debris

**DOI:** 10.1128/aem.01136-25

**Published:** 2025-07-24

**Authors:** Qi Zeng, Lili Jian, Songbiao Shi, Qiaoqiao Guo, Syed Raziuddin Quadri, Lijuan Long, Xinpeng Tian

**Affiliations:** 1 State Key Laboratory of Tropical Oceanography, South China Sea Institute of Oceanology, Chinese Academy of Sciences 74718 , Guangzhou, China; 2 Laboratory of Tropical Marine Bio-resources and Ecology, South China Sea Institute of Oceanology, Chinese Academy of Sciences 53042 , Guangzhou, China; 3 University of Chinese Academy of Sciences 521953, Beijing, China; 4 Department of Medical Laboratory Technology, Faculty of Applied Medical Sciences, Northern Border University 158223, Arar, Northern Borders, Saudi Arabia; Colorado School of Mines, Golden, Colorado, USA

**Keywords:** *Neptunicella plasticusilytica*, polyphasic characterization, plastic biodegradation, novel PET-degrading enzyme

## Abstract

Mangrove ecosystems are critical for coastal protection and biodiversity but are increasingly threatened by plastic pollution, particularly polyethylene terephthalate (PET). In this study, a novel marine bacterium, strain SCSIO 80796^T^, was isolated from PET debris collected from the mangrove in QiAo Island, Zhuhai, China. Using a polyphasic taxonomic approach, including 16S rRNA gene sequencing, genome-based comparisons (average nucleotide identity [ANI] 72.2%, digital DNA-DNA hybridization [dDDH] 19.0%, average amino acid identity [AAI] 70.6%), and phenotypic and chemotaxonomic analyses, the strain was classified as a novel species within the genus *Neptunicella*. It is proposed as *Neptunicella plasticusilytica* sp. nov. (type strain SCSIO 80796ᵀ = MCCC 1K08369^T^ = KCTC 92826^T^). Genomic analysis revealed that strain SCSIO 80796ᵀ encodes a novel PET-degrading enzyme, *Nm*Cut, which degrades PET and yields 105–120 µM of degradation products [terephthalic acid (TPA), mono(2-hydroxyethyl) terephthalate (MHET), bis(2-hydroxyethyl) terephthalate (BHET)] within 48 h at 60°C. *Nm*Cut exhibits both structural and evolutionary novelty, featuring a unique PET-binding module (PBM) absent in known PETases. PBM is characterized by a long, positively charged α-helix enriched in aromatic residues, forming a distinct substrate-interacting surface with potential as a transferable domain to enhance the efficiency of other plastic-degrading enzymes. This study not only expands the known diversity within *Neptunicella* but also highlights the potential of marine-derived microbes in addressing plastic pollution through biotechnological applications.

**IMPORTANCE:**

The discovery of *Neptunicella plasticusilytica* sp. nov. advances marine microbial ecology by revealing a novel species in the scarcely studied genus *Neptunicella*, which previously contained only one cultured representative. Isolated from plastic-polluted mangroves, this bacterium exemplifies microbial adaptation to anthropogenic habitats. Its functional uniqueness is underscored by a phylogenetically distinct polyethylene terephthalate (PET)-degrading enzyme (*Nm*Cut), forming an evolutionary clade separate from all known plastic-degrading enzymes. By integrating taxonomic discovery with functional genomics, this study bridges the gap between microbial diversity and biotechnological potential. The dual novelty of *N. plasticusilytica*—as a taxonomic addition and a source of evolutionarily unique enzymes—highlights the importance of exploring understudied environments to address global challenges like plastic pollution.

## INTRODUCTION

Polyethylene terephthalate (PET) is one of the most widely used plastics globally, with extensive applications in packaging and textiles (1). Its resistance to degradation has led to its accumulation in the environment, particularly in marine ecosystems, posing substantial threats to marine life and human health (2). Biodegradation, particularly through microbial enzymes, offers a promising solution to mitigate this issue (3). Therefore, it is important to excavate PET-degrading microorganisms from marine sources. Among various marine environments, mangrove environments not only act as biodiversity hotspots but also serve as effective traps for plastic pollutants due to tidal dynamics and extensive root systems that accumulate microplastics and debris (4, 5). These environments are relatively stable and anoxic, leading to extended residence times for pollutants. Besides, mangrove soils are rich in natural aromatic polymers such as lignin, cutin, and suberin, produced by accumulated plant biomass (5). Microbial communities in these ecosystems are exposed to a complex mixture of anthropogenic and natural polymers, providing evolutionary pressures that could select for enzymes capable of polymer degradation, even before widespread plastic pollution.

The phylum *Pseudomonadota* is well-known for encompassing numerous plastic-degrading bacteria, particularly within the genus *Pseudomonas* of the family *Pseudomonadaceae*. Species of *Pseudomonas* isolated from various environmental matrices have demonstrated the ability to degrade a wide range of plastics, including polyethylene, polypropylene, polyvinyl chloride, polystyrene, polyurethane, PET, polyethylene succinate, polyethylene glycol, and polyvinyl alcohol, with varying degrees of efficiency (6). In contrast, reports of plastic-degrading bacteria within the family *Alteromonadaceae* are relatively scarce. Among the 31 genera within *Alteromonadaceae*, only members of the genera *Glaciecola* and *Aestuariibacter* have been reported to degrade poly (3-hydroxybutyrate-co-3-hydroxyhexanoate) (7). The genus *Neptunicella* in this family was first described with the species *Neptunicella marina*, which was isolated from seawater. This bacterium is characterized as rod-shaped, Gram-stain-negative, aerobic, and motile with a polar flagellum (8). Despite its initial identification, the resource diversity within the genus *Neptunicella* remains largely unexplored, as *N. marina* is currently the sole species described. Although *N. marina* has been identified as part of the marine microbial ecosystem, its functional roles and potential applications remain poorly understood.

During our investigation of marine plastic-degrading bacteria, a novel species of the genus *Neptunicella* was isolated from PET debris collected from mangrove soil. This strain, designated SCSIO 80796ᵀ, was characterized as a new species, and its PET-degrading enzyme *Nm*Cut was also identified and characterized. The discovery of this new species not only expands the known diversity of the genus *Neptunicella* but also highlights its ecological significance in marine ecosystems. Furthermore, it provides a valuable microbial resource for the biodegradation of PET, addressing a critical challenge in plastic pollution management.

## MATERIALS AND METHODS

### Isolation, maintenance, and screening

Strain SCSIO 80796^T^ was isolated from plastic debris samples collected in 2022, from a mangrove in Qiao Island, China (22°25′51″N, 113°37′48″E). The samples were enriched in 25 mL minimal salts medium (MSM) with 1% PET powder under dark conditions at 28°C, 150 rpm for 30 days. After the enrichment period, the samples were diluted 50-fold, and 150 µL of the diluted suspension was spread onto MSM solid agar plates containing 1% PET powder. After being incubated at 28°C for 1 month in the dark, a pale yellow colony with a diameter of 1–1.5 mm was isolated. The purified strain SCSIO 80796^T^ was cultivated on 2216E medium and preserved in a 30% glycerol solution at −80°C for further studies.

### Cultural, morphological, and physiological properties

Cultural and morphological characteristics of cultures were assessed after 2 weeks on various agar media at 28°C, following Shirling and Gottlieb’s methodology (9). Growth temperature tolerance was assessed between 4°C and 55°C (4°C, 10°C, 20°C, 28°C, 37°C, 45°C, and 55°C) on 2216E agar plates. The pH tolerance (4.0–13.0 with intervals of 1.0 pH units) was evaluated in 2216E liquid medium at 28°C. The salt tolerance (0%, 1.0%, 2.0%, 3.0%, 4.0%, 5.0%, 6.0%, 7.0%, 8.0%, 9.0%, 10.0%, 12.0%, 15.0%, 18.0%, and 20% wt/vol NaCl) was determined using 2216E agar plates without NaCl at 28°C. Morphology was examined via transmission electron microscopy (JEM-100CX-II; JEOL) after a 3-day incubation. Cell motility was assessed using 2216E semisolid medium containing 0.4% agar. Gram-staining was assessed according to the steps of the standard Gram reaction combined with the KOH lysis test (10). Anaerobic growth was evaluated through the GasPak EZ Anaerobe Pouch system (BD). Biochemical reactions, including gelatin liquefaction, coagulation, and peptonization of milk, H_2_S production, nitrate reduction, and hydrolysis of cellulose, starch, Tweens (20, 40, 60, and 80), were observed following Tindall et al. (11). Catalase and oxidase activities were tested with hydrogen peroxide (vol/vol, 3%) and oxidase reagent (bioMérieux), respectively. Further biochemical characteristics and enzyme activities were analyzed using API ZYM (bioMérieux, Marcy-l'Étoile, France) and API 20NE kits (bioMérieux, Marcy-l'Étoile, France), and carbon and energy source utilization was evaluated with the Biolog GEN III system (Biolog, CA, USA).

### Chemotaxonomy

Chemotaxonomic analyses were performed on cultures grown at 28°C for 3 days on 2216E medium aerobically. Whole-cell fatty acids were analyzed using MIS Library software (Sherlock Version 6.1; MIDI database: TSBA6) following the manufacturer’s protocol (12). Quinone extractions and analyses were conducted according to Collins et al. (13). Polar lipids were extracted following Lechevalier’s method, analyzed by two-dimensional thin layer chromatography (14), and identified according to Minnikin et al*.* (15) using chloroform/methanol/water (65:25:4, vol/vol) for the first chromatographic phase and chloroform/methanol/acetic acid/water (80:18:12:5, vol/vol) for the second.

### Phylogenetic analyses

Genomic DNA was extracted using a DNA extraction kit (QIAGEN, Germany). The 16S rRNA gene was amplified using PCR with the recognized primer pair 27F (5′-AGAGTTTGATCCTGGCTCAG-3′) and 1492R (5′-GGTTACCTTGTTACGACTT-3′), and the resulting products were subsequently sequenced using the Sanger method. Sequence similarity analysis was conducted utilizing the EzBioCloud platform (https://www.ezbiocloud.net/). Multiple sequence alignments were executed with the CLUSTAL W (16). Phylogenetic analyses were conducted using the neighbor-joining (17), maximum-parsimony (18), and maximum-likelihood methods (19) as implemented in the MEGA 11 software with a bootstrap value of 1,000 resampling replicates (20).

To establish the prokaryote strain as a novel taxon, analyses of average nucleotide identity (ANI), digital DNA-DNA hybridization (dDDH), and average amino acid identity (AAI) were conducted. ANI was calculated with the ANI calculator (https://www.ezbiocloud.net/tools/ani) (21), dDDH was calculated using the genome-to-genome distance calculator 3.0 (https://ggdc.dsmz.de/ggdc.php) (22), and AAI was estimated by the AAI calculator tool (http://enve-omics.ce.gatech.edu) (23). Genome phylogenetic trees were constructed using RAxML (24) based on the 120 single-copy genes using the GTDB-Tk software toolkit (25).

### Genomic characterization and ecological distribution analysis

Genomic DNA was extracted using HiPure Bacterial DNA kits (Magen, Maryland, USA). A complete genome was sequenced using a PacBio RS II platform by Tianjin Biochip Corporation (Tianjin, China). A 5 µg of high-quality DNA was used to create 20 kb SMRTbell libraries, size-selected via Blue Pippin for longer inserts, and sequenced on a PacBio Sequel. Genome assembly was conducted using the HGAP4 Analysis Application software (26), with gene and amino acid sequences predicted by Prodigal (27). rRNA and tRNA predictions were performed with Barrnap and tRNAscan, respectively. eggNOG (28) and BLASTp against the Kyoto Encyclopedia of Genes and Genomes (KEGG) database were employed for genome functional annotation. Metabolic pathway reconstruction in a bacterium was achieved using KEGG Mapper (29), while secondary metabolism analysis was carried out with antiSMASH (30).

To investigate the global distribution and genomic diversity of the genus *Neptunicella*, a thorough investigation was carried out using public databases, such as NCBI GenBank, GBIF, IMG/MER, ENA Metagenomics, GTDB, and genomes from Earth’s Microbiomes to find the pure culture strains and metagenome-assembled genomes (MAGs) affiliated with this taxon. Full-length 16S rRNA gene sequences from the two available isolates were used as queries in BLASTn searches or Vsearch against the NCBI nucleotide database, EzBioCloud 16S rRNA reference data set, and Tara Oceans metagenomic catalog (31). Sequences with over 95% similarity were retained. Metadata for the matching sequences, including geographic coordinates and habitat descriptions, were extracted and cross-validated with the original publications. Geospatial visualization was conducted in R (v4.3.1) using the ggplot2 and sf packages.

### Prediction of PETase candidates using HMM and CLEAN

Initially, 17 previously reported high-efficiency plastic-degrading enzyme sequences were curated and aligned using Clustal Omega to ensure high-quality multiple sequence alignment (32). The alignment file was then converted to Stockholm format using the seqret tool (33), which is suitable for HMM profile construction. Using the HMMER package, an HMM profile was constructed to capture the conserved sequence motifs characteristic of plastic-degrading enzymes (34). The constructed HMM profile was used to search the proteome of strain SCSIO 80796^T^ for homologous sequences, utilizing the hmmsearch function of HMMER. This process identified candidate enzymes based on their alignment scores and statistical significance. The identified candidate enzymes were further annotated using the Swiss-Prot database and the CLEAN platform (35) (https://clean.platform.moleculemaker.org/configuration) to obtain information on their classification and function, thereby analyzing their potential roles in plastic degradation.

### Protein expression and purification

The predicted enzyme sequences were codon-optimized and synthesized by Guangzhou Tianyi Huiyuan Company. The genes were cloned into the pET22a (+) vector (Novagen) between the EcoRI and HindIII restriction sites with a C-terminal hexahistidine (6×His) tag. Recombinant plasmids were transformed into *Escherichia coli* BL21 (DE3) (Novagen). Transformed cells were grown in 2YT medium with 100 µg/mL ampicillin at 37°C until OD_600_ reached 0.6–0.8. Protein expression was induced with 1 mM IPTG (Sangon, Shanghai, China) at 16°C for 18 h. Cells were harvested by centrifugation at 5,000 × *g* for 10 min and resuspended in lysis buffer (20 mM Tris-HCl, pH 8.0, 500 mM NaCl, 5 mM imidazole). After sonication on ice and centrifugation at 10,000 × *g* for 30 min, the lysate was loaded onto Ni-NTA resin (Qiagen) equilibrated with binding buffer. The protein was washed with washing buffer and eluted with elution buffer (100 mM Tris-HCl, pH 8.0, 200 mM imidazole). Purified proteins were concentrated using an Amicon Ultra-15 device (10 kDa cut-off, Merck-Millipore). Protein purity was analyzed by SDS-PAGE, and protein concentrations were determined using a NanoDrop spectrophotometer at 280 nm.

### Enzymatic activity assay using *para*-nitrophenyl butyrate

The enzymatic activity of *Nm*Cut was evaluated using *para*-nitrophenyl butyrate (*p*NPB) (Aladdin, Shanghai, China) as a model substrate. Reactions were conducted in flat-bottom 96-well microplates in a total volume of 200 µL. Each well contained 195 µL of 20 mM Tris-HCl buffer (pH 8.0) supplemented with 0.25 mM *p*NPB and 5 µL of purified *Nm*Cut (1 µg/mL; protein concentration determined using a NanoDrop 2000 spectrophotometer, Thermo Scientific, USA, at 280 nm). Heat-inactivated enzyme (boiled at 100°C for 15 min) was used as a negative control. Reactions were incubated at 60°C for 10 min, and the release of p-nitrophenol (*p*NP) was monitored at 405 nm using an EnSight multimode microplate reader (PerkinElmer Inc., Spokane, WA, United States). To determine the optimal reaction temperature and pH, enzyme activity was evaluated at temperatures ranging from 20°C–70°C and pH values ranging from 4.0 to 10.0. The effect of metal ions (e.g., Ca²^+^, Mg²^+^, Zn²^+^) on *Nm*Cut activity was assessed by adding metal ions to the reaction mixture at final concentrations of 1 and 10 mM. Thermal stability was assessed by pre-incubating *Nm*Cut at 60°C for various durations (0, 2, 4, 6, 8, 10, 12, 24, 36, and 48 h), followed by the standard *p*NPB activity assay to determine residual enzymatic activity. All assays were performed in triplicate. Data were expressed as mean ± standard deviation (SD).

### PETase activity assays

PET-degrading activity of the purified *Nm*Cut was assessed using both PET powder (PETP, 10.3% crystallinity; Goodfellow, Huntingdon, UK) and amorphous PET films (PETF, 7.1% crystallinity; Goodfellow, Huntingdon, UK). Enzymatic reactions were conducted in 1.5 mL microcentrifuge tubes containing 1 µM purified *Nm*Cut (protein concentration determined using a NanoDrop 2000 spectrophotometer, Thermo Scientific, USA, at 280 nm), 15 mg PET substrate, and 1 mL of 20 mM potassium phosphate buffer (pH 8.0). Reaction mixtures were incubated at 60°C for up to 48 h with agitation (900 rpm). Heat-inactivated enzyme (boiled at 95°C for 15 min) served as the negative control. At defined time intervals (8, 12, 24, and 48 h), aliquots of the supernatant were collected and filtered (0.22 µm). Bis(2-hydroxyethyl) terephthalate (BHET), mono(2-hydroxyethyl) terephthalate (MHET), and terephthalic acid (TPA) were quantified using high-performance liquid chromatography (HPLC) on an Agilent 1200 system with an SB C-18 column (5 µm, 4.6 × 150 mm, Agilent). The mobile phase consisted of solvent A (water with 0.1% formic acid) and solvent B (methanol), using the following gradient: 0–5 min, 70:30 A/B; 5–20 min, 20:80 A/B; 20–23 min, 100% B; 23–30 min, 70:30 A/B. The flow rate was 1 mL/min, and detection was performed at 240 nm. Peak identities were confirmed and quantified using commercial standards for BHET, MHET, and TPA. All assays were performed in triplicate. Data were expressed as mean ± standard deviation (SD). To assess morphological changes on PET films, residual PETF was washed with 1 M PBS (pH 7.4), air-dried at 35°C, mounted on aluminum stubs, sputter-coated with gold, and examined under a Hitachi S-4300 scanning electron microscope (SEM) at 10 kV.

### Structural and phylogenetic analysis of *Nm*Cut

Phylogenetic analysis was conducted using representative PETases and cutinases retrieved from NCBI and UniProt. Multiple sequence alignment was performed using MUSCLE (36), and a maximum likelihood tree was constructed in MEGA X with 1,000 bootstrap replicates. The 3D structure of *Nm*Cut was predicted using AlphaFold3 (37) and validated with PROCHECK (38). Structural comparisons with known PETases (e.g., *Is*PETase [39], LCC [40], TfCut2 [41]) were conducted using PyMOL, and root mean square deviation (RMSD) values were calculated to assess structural divergence. Electrostatic surface potentials were calculated using APBS (42) in PyMOL. Molecular docking of a PET trimer was performed using AutoDock Vina (43), with the active site defined around the catalytic triad. Binding affinities and key interacting residues were analyzed with Protein-Ligand Interaction Profiler (44).

## RESULTS AND DISCUSSION

### Phenotypic and chemotaxonomic characteristics of strain SCSIO 80796ᵀ

To investigate the biological characteristics of the PET-degrading bacterium SCSIO 80796ᵀ, we first assessed its phenotypic traits, biochemical behaviors, and chemotaxonomic markers in comparison with closely related species. Strain SCSIO 80796ᵀ was isolated from MSM agar plates supplemented with PET as the sole carbon source. On 2216E agar, after 3 days of incubation, the strain formed creamy-white, opaque, smooth, convex, and circular colonies. The cells are aerobic, Gram-stain-negative rods, measuring 1.0–2.0 μm in length and 0.5–1 μm in width, and exhibit motility via a single polar flagellum (Fig. S1). The strain grows optimally at 28°C, pH 7.0, and 0.5%–2% NaCl, tolerating wide environmental ranges (4°C–40°C, pH 6.0–9.0, and up to 12% NaCl).

Biochemical profiling revealed distinct differences from the closest known species, *Neptunicella marina* KCTC 52335ᵀ. Strain SCSIO 80796^T^ hydrolyzes Tweens 20, 40, 60, and 80 and tests positive for *β*-galactosidase and *β*-glucuronidase but lacks activity for enzymes such as trypsin and *α*-chymotrypsin (Table S1). In substrate utilization tests, it assimilated *L*-arabinose, *myo*-inositol, and acetoacetic acid—substrates not utilized by *N. marina*. Chemotaxonomic analysis showed major fatty acid composition of strain SCSIO 80796^T^ is C_16:0_ (21.7%), C_16:1_ *ω*7*c*/*ω*6*c* (23.9%), and C_18:1_ *ω*7*c*/*ω*6*c* (9.0%) (Table S2). The predominant respiratory quinone is Q-8. The polar lipid profile of strain SCSIO 80796ᵀ includes phosphatidylethanolamine (PE), phosphatidylglycerol (PG), one unidentified phospholipid (PL), and two unidentified lipids (L), similar to but less complex than that of *N. marina* (Fig. S2). These collective characteristics support the classification of strain SCSIO 80796^T^ within the genus *Neptunicella*.

### Phylogeny and genomic delineation of strain SCSIO 80796ᵀ

**Fig 1:**
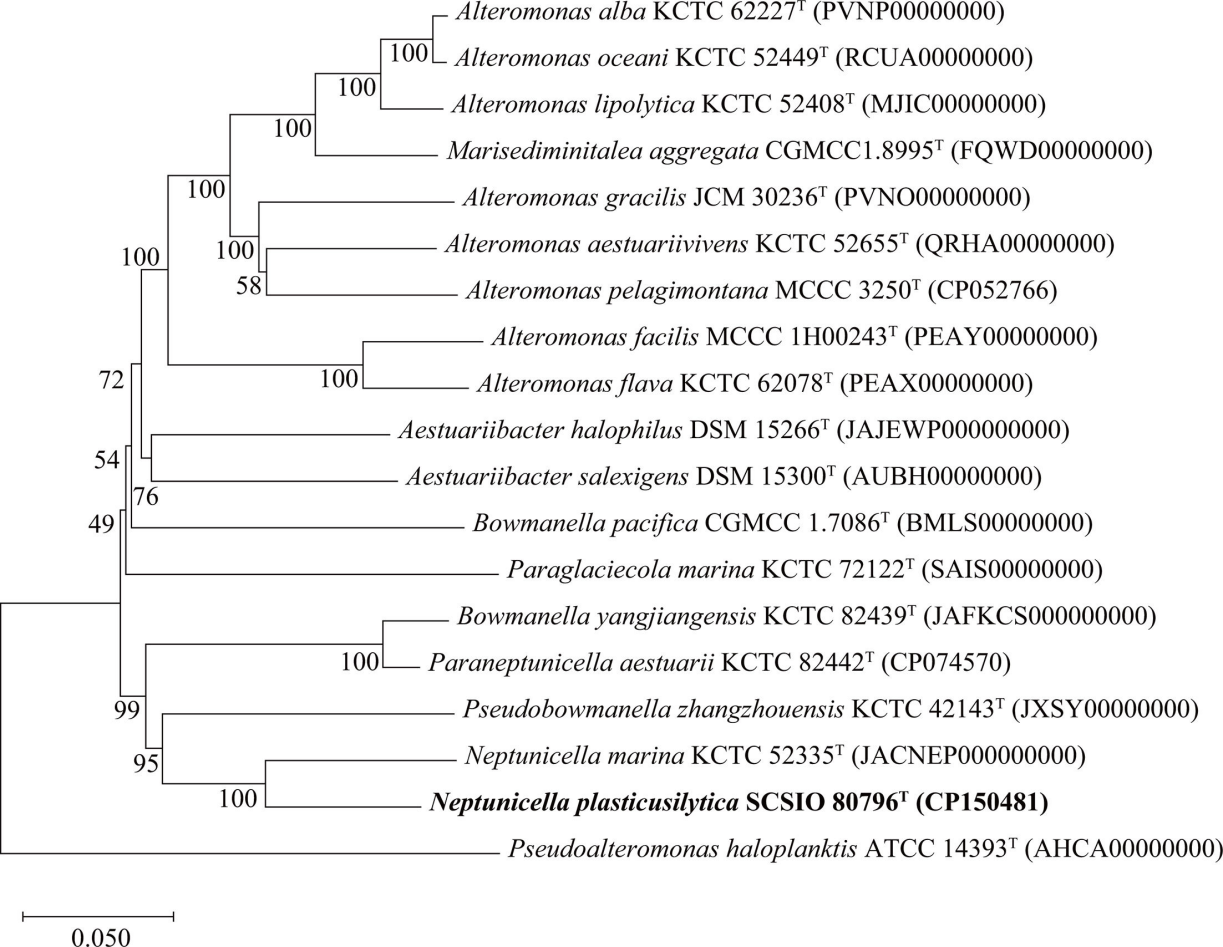
Phylogenetic analysis based on genome sequences of strain SCSIO 80796^T^ with its closest related taxa. *Pseudoalteromonas haloplanktis* ATCC 14393^T^ was used as an outgroup. Bar: 0.05 substitutions per nucleotide position.

In order to determine the taxonomic affiliation of strain SCSIO 80796ᵀ, both 16S rRNA gene sequencing and whole-genome comparisons were conducted. The nearly full-length 16S rRNA gene sequence of SCSIO 80796^T^ (1,418 bp; GenBank accession number PP212059) showed the highest similarity to *N. marina* KCTC 52335^T^ (95.4%). Phylogenetic analysis based on the 16S rRNA gene sequences, using neighbor-joining (Fig. S3), maximum-likelihood (Fig. S4), and maximum-parsimony (Fig. S5) methods, indicated that strain SCSIO 80796^T^ clustered with its closest relatives and clearly formed one distinct lineage within the genus *Neptunicella*. The genome of SCSIO 80796^T^ comprises a single circular chromosome of 4,456,699 bp (accession number CP150481), encoding 3,954 protein-coding genes, 12 rRNAs, and 57 tRNAs (GC content: 46.0%). No plasmids were identified (Table S3). The genome phylogenetic tree (Fig. 1), constructed based on 120 conserved marker genes, also demonstrated that strain SCSIO 80796^T^ forms a distinct lineage within the genus *Neptunicella*, consistent with the 16S rRNA gene phylogenetic position. Genomic comparisons further supported this distinction. ANI between SCSIO 80796^T^ and *N. marina* was 72.2%, with dDDH and AAI values of 19.0% and 70.6% with *N. marina* KCTC 52335^T^, respectively (Table S4), all well below the standard species delineation thresholds (ANI ≥ 95%, dDDH ≥ 70%, AAI ≥ 95%) (45). These data confirm that SCSIO 80796^T^ represents a novel species within the genus *Neptunicella*.

**Fig 2:**
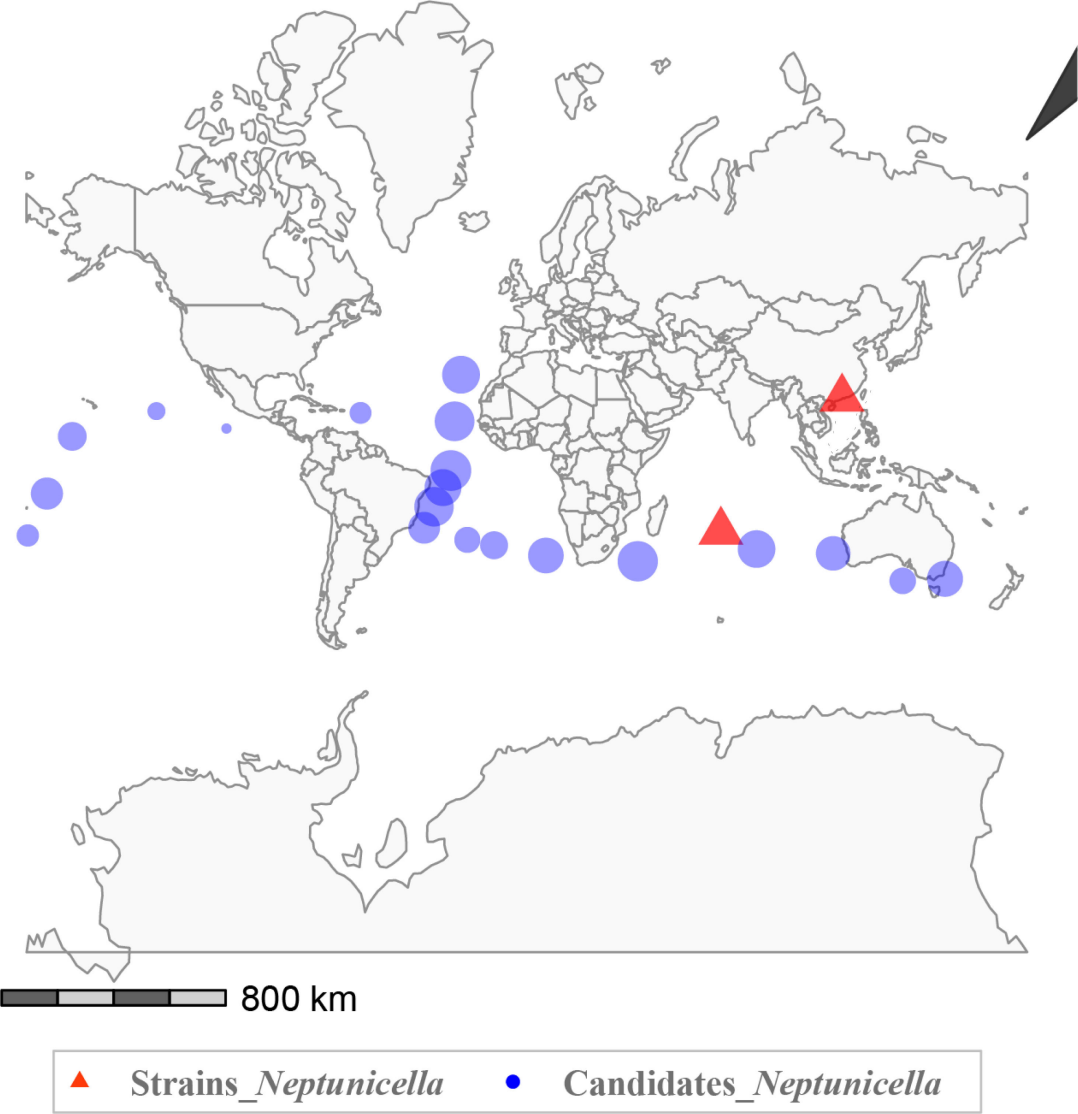
Global distribution of *Neptunicella* species. Red triangles represent the pure culture strains, and blue circles indicate candidate taxa. Circle size corresponds to relative abundance. The map was generated in R using the rnaturalearth package.

To assess the ecological relevance of this lineage, we examined public databases and found that the genus *Neptunicella* is poorly represented, with only one isolated strain and no MAGs identified (Fig. 2). The cultured strains (indicated by red triangles) include one previously isolated from surface seawater in the Indian Ocean and the other obtained from plastic debris in mangrove sediment in this study. Vsearch significant differences analysis based on their 16S rRNA sequences (with over 95% similarity) identified six different matches that were discovered in 60 samples from the Tara Oceans database. This indicates that *Neptunicella* is predominantly distributed in deep-water layers of the Atlantic Ocean (depth range: −3,000 to −4,000 m), though it exhibits a limited presence in other oceanic regions. Notably, *Neptunicella* lineages were found to span a broad longitudinal range and exhibited a latitudinal enrichment in equatorial and warm oceanic areas, with maxima localized near a plateau in the North Pacific (10.0927°N, 99.2462°W) and minima in a seamount in the Indian Ocean (−31.16°S, 110.18°E). Overall, these findings emphasize the extremely limited biological resources related to the genus *Neptunicella* (Table S5).

### Genome mining for PET-degrading enzymes

To explore the molecular basis of PET degradation in strain SCSIO 80796ᵀ, we constructed HMM profiles using 17 well-known plastic-degrading enzyme sequences (Table S6) and employed HMMER to search the complete genome sequence of strain SCSIO 80796^T^. This analysis identified 14 candidate genes with significant similarity to known plastic-degrading enzymes. Comparative analysis with the Swiss-Prot database and predictions using CLEAN revealed several candidate enzymes annotated as peptidases, esterases, and cutinases (Table 1). Each of these enzymes was heterologously expressed and purified in *E. coli* to assess their activity. Among them, only the enzyme corresponding to gene SCSIO80796_2563 exhibited PET-degrading activity, and it was subsequently named *Nm*Cut. *Nm*Cut with the second-highest HMM score (Table S7) was annotated in the Swiss-Prot database as a leaf-branch compost cutinase, EC 3.1.1.1, a carboxylesterase predicted by CLEAN. The original leaf-branch compost cutinase (46) from the metagenome of leaf-branch compost is recognized as a highly efficient PET-degrading enzyme and serves as the template for several engineered variants like LCC^ICCG^. These results suggest that *Nm*Cut may share mechanistic or structural features with this highly active class of PETases.

**TABLE 1:** Candidate PET-degrading enzymes identified from the genome of strain SCSIO 80796ᵀ

Gene ID	Swiss-Prot annotation	CLEAN prediction	PETase activity
SCSIO80796_2283	Dipeptidyl aminopeptidase BIII	EC 3.4.19.1—acylaminoacyl-peptidase	No
SCSIO80796_2563	Leaf-branch compost cutinase	EC 3.1.1.1—carboxylesterase	Yes (*Nm*Cut)
SCSIO80796_0503	Dipeptidyl aminopeptidase 4	EC 3.4.14.5—dipeptidyl-peptidase IV	No
SCSIO80796_2105	Esterase YbfF	EC 3.1.2.1—acetyl-CoA hydrolase	No
SCSIO80796_1158	Dipeptidyl aminopeptidase 4	EC 3.1.1.72—acetylxylan esterase	No
SCSIO80796_3201	Putative hydrolase fragment YghX	EC 3.1.1.45—carboxymethylenebutenolidase	No
SCSIO80796_0871	Macro domain-containing protein	EC 3.1.1.106—O-acetyl-ADP-ribose deacetylase	No
SCSIO80796_3782	Carboxylesterase 2	EC 3.1.1.1—carboxylesterase	No
SCSIO80796_0232	Acyl-coenzyme A thioesterase 2	EC 3.1.1.1—carboxylesterase	No
SCSIO80796_2388	Proline iminopeptidase	EC 3.4.11.5—prolyl aminopeptidase	No
SCSIO80796_1097	S-formylglutathione hydrolase YeiG	EC 3.1.2.12—S-formylglutathione hydrolase	No
SCSIO80796_1452	Probable carboxylic ester hydrolase LipM	EC 3.1.1.1—carboxylesterase	No
SCSIO80796_1339	Putative 2-succinyl-6-hydroxy-2,4-cyclohexadiene-1-carboxylate	EC 3.1.1.1—carboxylesterase	No

### Biochemical properties and PET-degrading activity of *Nm*Cut

**Fig 3:**
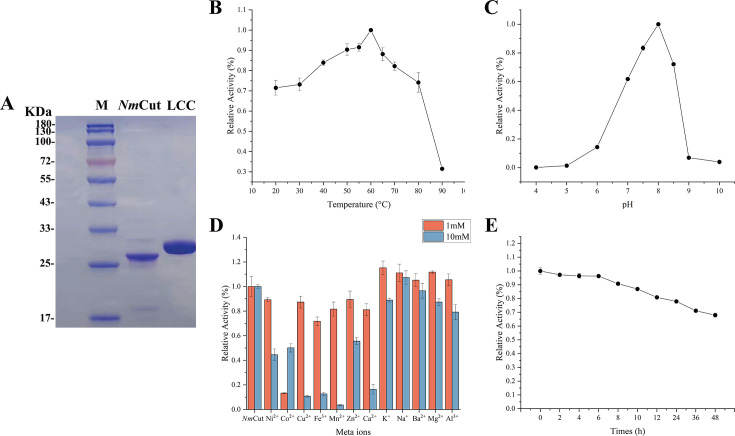
Catalytic properties of *Nm*Cut. (**A**) SDS-PAGE gel showing the protein purity of *Nm*Cut. (**B**) Optimal reaction temperature of *Nm*Cut. (**C**) Optimal reaction pH of *Nm*Cut. (**D**) Effect of metal ions on the activity of *Nm*Cut. (**E**) Thermal stability of *Nm*Cut at 60°C. All assays were conducted in triplicate using p-nitrophenyl butyrate (pNP-butyrate) as the substrate. Data are shown as mean ± SD (*n* = 3).

To evaluate the catalytic potential of *Nm*Cut, we first characterized its biochemical properties using *p*-nitrophenyl acetate (*p*NPB) as a model substrate. *Nm*Cut comprises 277 amino acid residues and has a molecular weight of 28 kDa (Fig. 3A). The enzyme exhibited maximum activity at 60°C (Fig. 3B) and pH 8 (Fig. 3C), conditions that were subsequently adopted for downstream PET degradation assays. The presence of metal ions, including K^+^, Na^+^, Ba²^+^, Mg²^+^, and Al³^+^, enhanced the ester bond-degrading activity of *Nm*Cut (Fig. 3D). Furthermore, *Nm*Cut demonstrated notable thermal stability, retaining 68% of its initial activity after 48 h incubation at 60°C (Fig. 3E).

**Fig 4:**
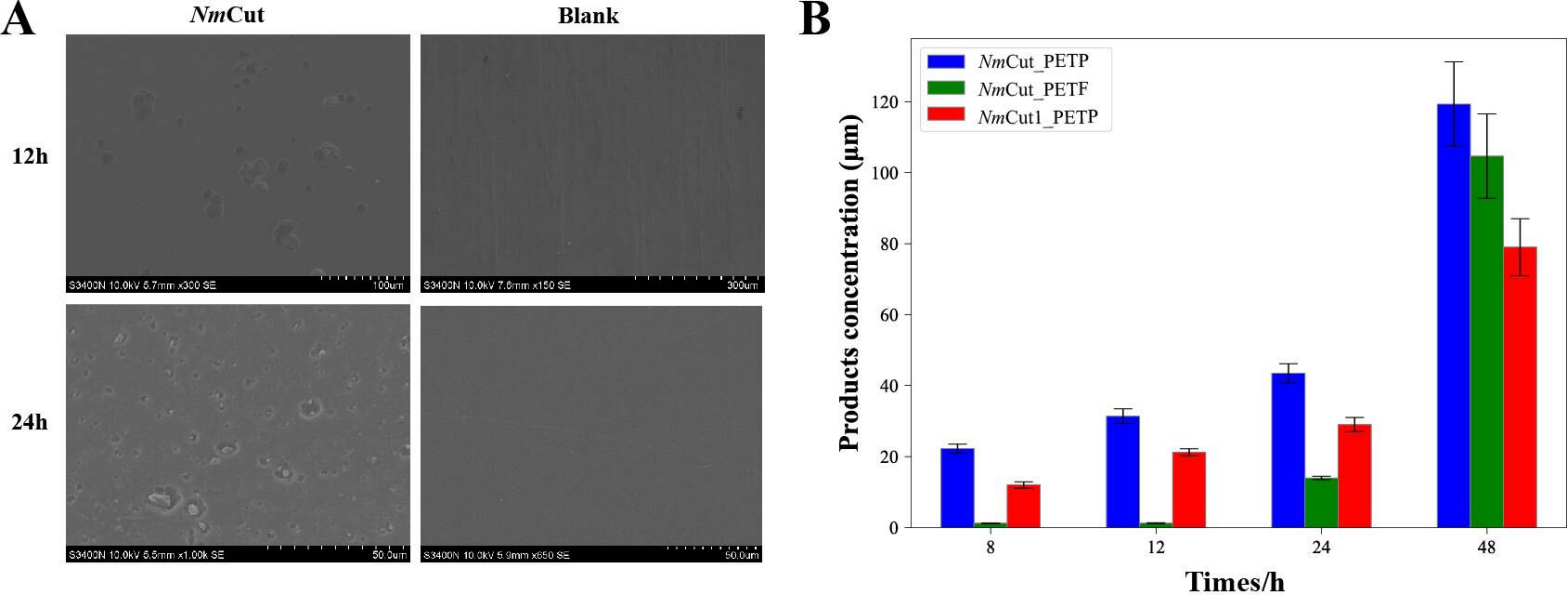
PET degradation activity of *Nm*Cut. PET (15 mg) was incubated with 1 µM *Nm*Cut in pH 8.0 buffer for 24 h at 60°C. (**A**) SEM images showing the surface morphology of PET degradation by *Nm*Cut at 60°C. (**B**) Quantification of degradation products (TPA, MHET, BHET) released by *Nm*Cut and *Nm*Cut1 (*Nm*Cut without helix) in pH 8.0 potassium phosphate buffer over 48 h at 60°C. PETP is 11.3% crystallinity PET powder, and PETF is 7.1% crystallinity PET film. Data are shown as mean ± SD (*n* = 3).

After optimizing reaction conditions, we assessed the degradation activity of *Nm*Cut at pH 8 and 60°C against two types of PET substrates: PET powder with 11.3% crystallinity (PETP) and low-crystallinity amorphous PET films with 7.1% crystallinity (PETF). SEM analysis revealed that *Nm*Cut effectively degraded PET films, producing significant surface erosion and pitting after 24 h (Fig. 4A). Quantification of the degradation products by HPLC confirmed the formation of TPA, MHET, and BHET, with cumulative product concentrations reaching 105–120 µM after 48 h (Fig. 4B; standard curves shown in Fig. S6). Interestingly, *Nm*Cut displayed higher activity against PETP than PETF, despite the former’s higher crystallinity. This suggests that substrate morphology, particularly the larger specific surface area of PET powder, plays a more critical role in enzymatic accessibility and hydrolysis than crystallinity alone (47). The continuous increase in degradation product concentrations over the 48 h period indicates that *Nm*Cut maintained its PET-degrading activity at 60°C for an extended duration (Fig. 4B). However, in comparison with the benchmark PET hydrolase LCC, *Nm*Cut exhibited relatively modest PET-degrading activity (Fig. S7), suggesting that future protein engineering efforts may be necessary to enhance its performance.

### Phylogenetic and structural analysis of *Nm*Cut

To investigate the evolutionary and structural characteristics of *Nm*Cut, we conducted phylogenetic analysis, multiple sequence alignment, structural modeling, and molecular docking.

**Fig 5:**
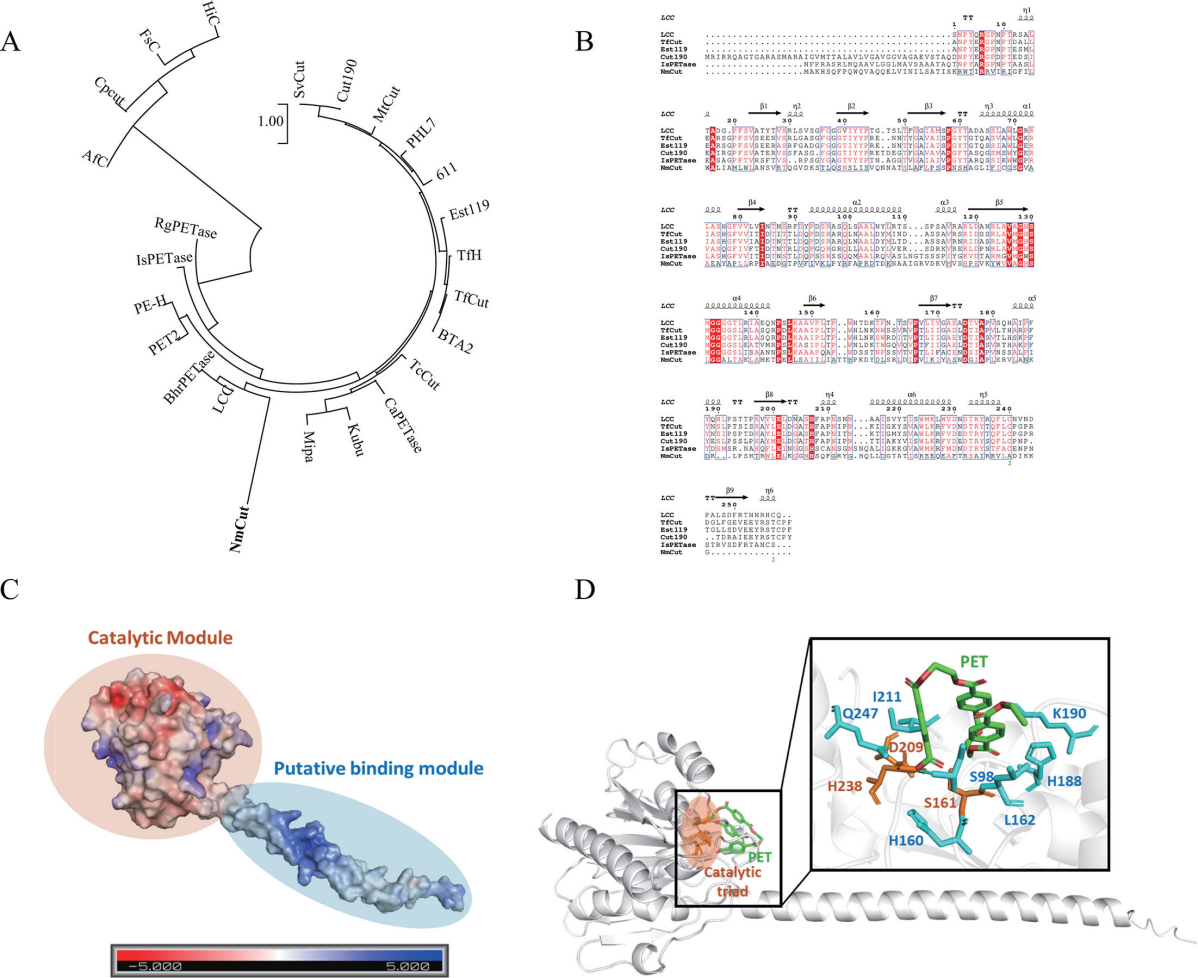
Structural and phylogenetic characterization of *Nm*Cut. (**A**) Maximum likelihood (ML) phylogenetic tree based on the amino acid sequence of *Nm*Cut and other known PET-degrading enzymes. (**B**) Multiple sequence alignment of *Nm*Cut with representative PETases. (**C**) Electrostatic surface representation of *Nm*Cut, showing a modular organization: a catalytic module (CM, orange) and a putative binding module (PBM, blue). Color scale indicates surface potential from −5.0 (red) to +5.0 (blue) kcal/mol·e. (**D**) Molecular docking model of *Nm*Cut with a PET trimer (green). The catalytic triad (Ser161, Asp209, His237) is shown in orange; predicted mutation sites enhancing PET interaction are indicated in blue.

We conducted BLASTP analysis against the NCBI non-redundant (nr) database. The top hits were annotated as hypothetical proteins or generic *α*/*β*-hydrolases, with the highest sequence identity reaching only 64.2%, supporting its classification as a putative orphan enzyme. Besides, phylogenetic analysis based on representative PET-degrading enzymes shows that *Nm*Cut forms a distinct clade positioned between fungal and bacterial PETases, suggesting it represents a novel PET-degrading enzyme (Fig. 5A). The mangrove ecosystem likely constitutes a significant reservoir of plastic-degrading enzymes, driven by persistent selective pressures from anthropogenic plastic accumulation in coastal sediments. Besides, mangrove environments are enriched with plant-derived compounds such as lignin, cutin, and other aromatic polyesters, which may act as natural analogs to PET (48). The presence of these structurally similar substrates could promote the evolution or retention of PETases. Compared to other marine ecosystems, such as the deep sea, where such terrestrial inputs are minimal, mangroves may provide a more favorable setting for the functional adaptation of PETases (49).

Multiple sequence alignment with representative PET-degrading enzymes revealed that *Nm*Cut adopts a classical *α*/*β*-hydrolase fold and contains a conserved catalytic triad (Ser161-Asp209-His237), characteristic of PETases (Fig. 5B). We then used AlphaFold3 to predict the tertiary structure of *Nm*Cut. The predicted model was validated using PROCHECK, confirming its stereochemical soundness (Fig. S8). To further examine its structural distinctiveness, we conducted structural superposition of *Nm*Cut with well-characterized PETases, including *Is*PETase, LCC, and TfCut2, using PyMOL. The resulting RMSD values ranged from 2.1 to 24.9 Å, indicating significant structural divergence, particularly outside the catalytic core. These observations strongly support the novelty of *Nm*Cut among known PETases.

To better understand how *Nm*Cut interacts with PET, we analyzed the predicted protein structure using electrostatic surface potential mapping and molecular docking simulations. AlphaFold3-based tertiary structure prediction revealed that *Nm*Cut adopts a modular organization (Fig. 5C), comprising a catalytic module (CM) with the canonical *α*/*β*-hydrolase fold, and an extended helical region we propose as a putative binding module (PBM). Electrostatic surface potential analysis via APBS showed that the PBM is enriched in positively charged residues and aromatic residues, forming a protruding domain ideal for substrate adsorption and interaction with PET surfaces. These structural and physicochemical features suggest that the PBM may play a key role in substrate recognition. To verify our hypothesis, we generated a PBM-deleted variant and observed a decrease in enzymatic activity toward PET, confirming the functional importance of this helical domain (Fig. 4B). Finally, molecular docking simulations showed PET oligomer binding near the catalytic triad (Fig. 5D), with a binding free energy of –6.88 kcal/mol. Residues involved in substrate interaction, such as K190, H188, H160, and Q247, were identified as promising targets for mutagenesis to enhance catalytic efficiency.

### Conclusions

In this study, strain SCSIO 80796ᵀ was isolated from PET debris in a mangrove environment and characterized as a novel species within the genus *Neptunicella*. Phylogenetic analysis based on the 16S rRNA gene and genome showed close relatedness to *N. marina*, and genomic indices (ANI, dDDH, AAI) confirmed its species-level distinction. Phenotypic and chemotaxonomic differences, including polar lipid composition, further supported its classification as a new species. Accordingly, strain SCSIO 80796ᵀ is proposed as a novel species, for which the name *Neptunicella plasticusilytica* sp. nov. is proposed. Additionally, a novel PET-degrading enzyme, *Nm*Cut, was discovered in strain SCSIO 80796ᵀ. *Nm*Cut exhibits optimal activity at 60°C and pH 8 and demonstrates notable thermal stability, which retains 68% of its activity after 48 h at 60°C. The enzyme effectively degrades PET, producing 105–120 µM degradation products (TPA, MHET, BHET) within 48 h. *Nm*Cut exhibits a structurally and evolutionarily novel feature, featuring a functionally significant PBM that may serve as a transferable binding module to enhance the performance of other plastic-degrading enzymes. This modularity underscores its potential as a scaffold for enzyme engineering in plastic biodegradation applications. The discovery of the new species and the PET-degrading enzyme *Nm*Cut not only contributes to our understanding of the microbial diversity within the *Neptunicella* genus but also underscores its potential in biotechnological applications. These findings offer valuable insights into the use of marine-derived microbes for addressing PET pollution and lay the groundwork for future studies focusing on the optimization of *Nm*Cut and its broader application in plastic biodegradation.

## SUPPLEMENTAL MATERIAL

Supplemental material aem.01136-25-s0001.pdf Figures S1 to S8; Tables S1 to S7.

ASM does not own the copyrights to Supplemental Material that may be linked to, or accessed through, an article. The authors have granted ASM a non-exclusive, world-wide license to publish the Supplemental Material files. Please contact the corresponding author directly for reuse.
