# Supplementary material for: Identified Neptunicella plasticusilytica sp. nov. and its novel PET-degrading enzyme derived from mangrove plastic debris
Source: Appl Environ Microbiol. 2025 Jul 24;91(8):e01136-25. doi: 10.1128/aem.01136-25 (PMC12366299; doi:10.1128/aem.01136-25)
Supplement: Supplemental material — Figures S1 to S8; Tables S1 to S7. [file aem.01136-25-s0001.pdf]

Identified *Neptunicella plasticusilytica* sp. nov. and its Novel PET-Degrading Enzyme derived from Mangrove Plastic Debris

Qi Zeng[1,2,3], Lili Jian[1,3], Songbiao Shi[1,2], Qiaoqiao Guo[1,2], Syed Raziuddin Quadri[4], Lijuan Long[1,2], Xinpeng Tian[1,2]*

[1]State Key Laboratory of Tropical Oceanography, South China Sea Institute of Oceanology, Chinese Academy of Sciences, Guangzhou 510301, China.

[2]CAS Key Laboratory of Tropical Marine Bio-resources and Ecology, South China Sea Institute of Oceanology, Chinese Academy of Sciences, Guangzhou 510301, China.

[3]University of Chinese Academy of Sciences, Beijing 100049, China.

[4]Department of Medical Laboratory Technology, Faculty of Applied Medical Sciences, Northern Border University, Arar-91431, Northern Borders, Kingdom of Saudi Arabia.

***Corresponding authors and linkage address:**

Xinpeng Tian

Tel / Fax: +86 20 89023378

E-Mail: xinpengtian@scsio.ac.cn

Running title: *Neptunicella plasticusilytica* sp. nov.

Category: New species –*Neptunicella plasticusilytica* sp. nov.

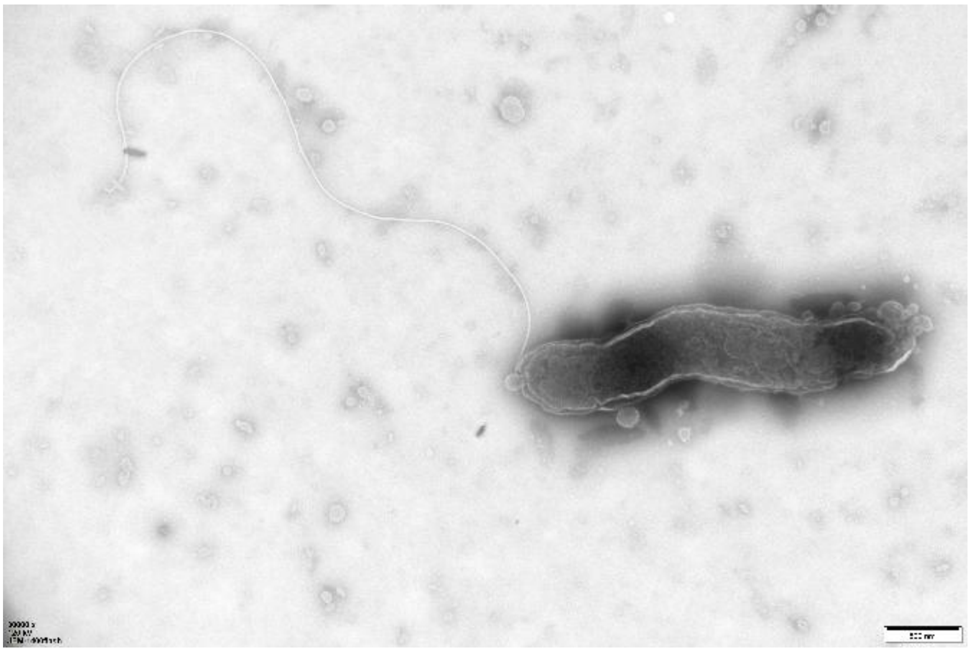

Figure S1. Transmission electron micrographs showing the cell morphology of strain SCSIO 80796^T after incubation on 2216E agar medium for 3 days at 28°C.

Table S1. Distinguished phenotypic characteristics of *Neptunicella plasticusilytica* SCSIO 80796^T and its phylogenetically relative. Both strains were negative for the hydrolysis of starch, cellulose, casein, reduction of nitrates to nitrites or nitrogen, production of indole, H_2S and coagulation and peptonization of milk, and positive for aesculin, oxidase and catalase. In API 20NE tests, they were positive for arginine dihydrolase, urease and aesculin hydrolysis. In the API ZYM kits, they were positive for alkaline phosphatase, Esterase (C_4), Esterase lipase (C_8), lipase (C_{14}), leucine arylamidase, valine arylamidase, cystine arylamidase, acid phosphatase, naphtol-AS-BI-phosphohydrolase, *a*-galactosidase, *α*-Glucosidase, *β*-Glucosidase, *N*-Acetyl-*β*-glucosaminidase.

Characteristic	*Neptunicella plasticusilytica* SCSIO 80796^T	*Neptunicella marina* KCTC 52335^T
Temperature (°C)	4–40	4–40
pH	6–9	6–9
NaCl (%, *w*/*v*)	0–12	0–12
Motile	+	+
β-galactosidase	+	−
β-glucuronidase	+	−
trypsin	−	+
α-chymotrypsin	−	+
α-mannosidase	−	+
β-fucosidase	−	+
L-arabinose	+	−
malic acid	−	+
myo-inositol	+	−
acetoacetic acid	+	−

Table S2 Fatty acid comparison of strains SCSIO 80796^T and the closely related neighbors. Strains: 1, SCSIO 80796^T; 2, *Neptunicella marina* KCTC 52335^T. All data were obtained from this study. Values are percentages of total fatty acids. The major fatty acids (greater than 10%) are shown in bold. TR, less than 1%.

Fatty acid	1	2
Saturated		
$C_{12:0}$	4.70	3.96
$C_{13:0}$	1.74	1.18
$C_{14:0}$	2.04	1.47
$C_{16:0}$	**21.69**	**21.87**
$C_{17:0}$	5.70	5.57
$C_{18:0}$	TR	1.04
Unsaturated		
$C_{15:1}$ $\omega 8c$	TR	TR
$C_{17:1}$ $\omega 8c$	4.46	4.86
Branched		
Iso-$C_{14:0}$	TR	TR
Iso-$C_{16:0}$	1.04	2.25
Hydroxy		
$C_{10:0}$ 3-OH	2.9	5.84
$C_{12:0}$ 3-OH	5.03	5.00
$C_{16:0}$ 3-OH	TR	1.13
Summed features*		
$C_{16:1}$ $\omega 7c/\omega 6c$	**23.90**	**17.45**
$C_{18:1}$ $\omega 7c/\omega 6c$	**8.98**	**15.62**

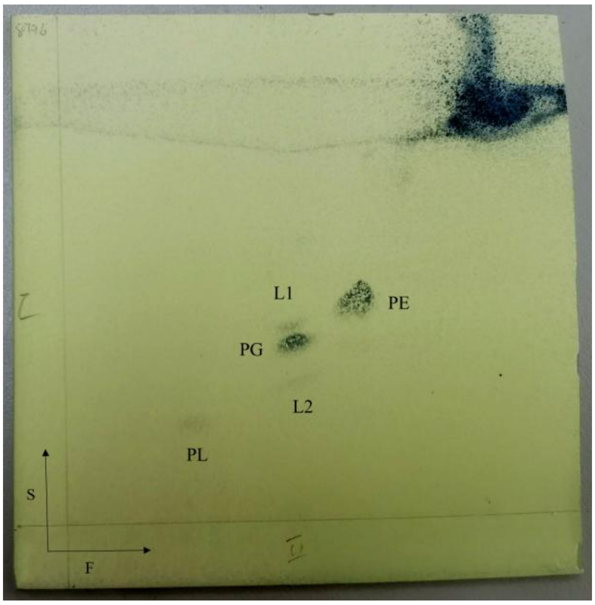

Fig. S2 Two-dimensional thin-layer chromatography of polar lipids of strains SCSIO 80796^{T}. The first direction developed in chloroform: methanol: water (65:25:4, v/v/v), and the second in chloroform: methanol: acetic acid: water (80:12:15:4, v/v/v/v). PE, phosphatidylethanolamine; PG, phosphatidylglycerol; PL, unidentified phospholipid; L, unidentified lipid; F-first dimension of TLC; S- second dimension of TLC.

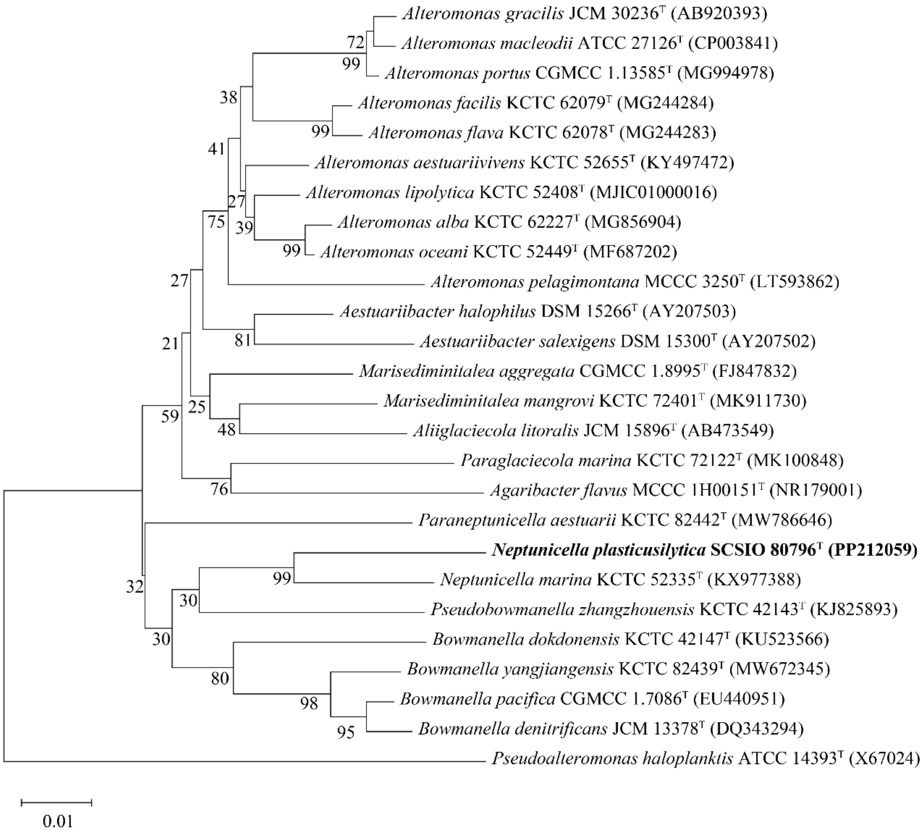

Fig. S3 Neighbour-joining phylogenetic tree based on nearly complete 16S rRNA gene sequences of strain SCSIO 80796^{T} with its closest related taxa. *P. haloplanktis* ATCC 14393^{T} was used as an outgroup. Bar, 0.01 substitutions per nucleotide position.

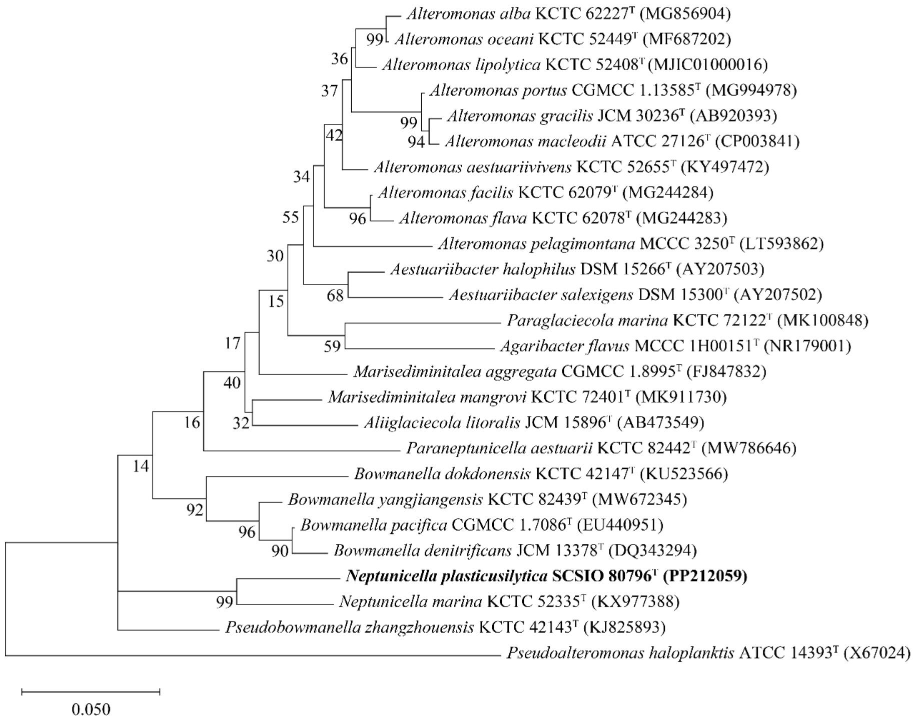

Fig. S4 The maximum-likelihood phylogenetic tree based on nearly complete 16S rRNA gene sequences of strain SCSIO 80796^T with its closest related taxa. *P. haloplanktis* ATCC 14393^T was used as an outgroup. Bar, 0.050 substitutions per nucleotide position.

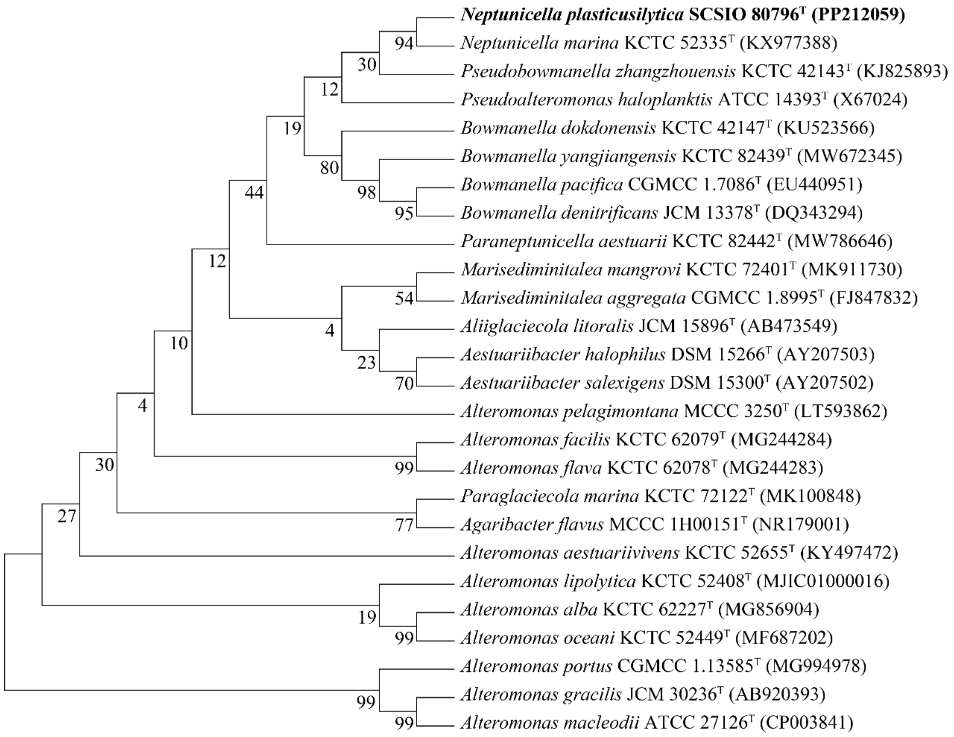

Fig. S5 The maximum-parsimony phylogenetic tree based on nearly complete 16S rRNA gene sequences of strain SCSIO 80796^T with its closest related taxa. *P. haloplanktis* ATCC 14393^T was used as an outgroup.

Table S3. General genome features of strains SCSIO 80796^T and *N. marina* KCTC 52335^T.

Strain	Genome size (Mbp)	G+C content (%)	Contigs	Genes
SCSIO 80796^T	4.46	46.0	1	3954
KCTC 52335^T	4.14	43.5	46	3770

Table S4 Results of ANIb, AAI and dDDH between genomes of strain SCSIO 80796^T and its most closely related species.

**Strains**	**SCSIO 80796^T**		
	**ANI (%)**	**dDDH (%)**	**AAI (%)**
Neptunicella marina KCTC 52335^T	72.21	19.00%	70.60%
Alteromonas aestuariivivens KCTC 52655^T	68.87	21.80%	59.29%
Pseudobowmanella zhangzhouensis KCTC 42143^T	69.64	19.20%	62.54%
Alteromonas pelagimontana MCCC 3250^T	68.68	25.10%	58.91%
Alteromonas alba KCTC 62227^T	68.99	23.10%	57.41%

Table S5. Descriptions of *Neptunicella plasticusilytica* sp. nov.

Species name	*Neptunicella plasticusilytica*
Specific epithet	*plasticusilytica*
Species status	sp. nov
Species etymology	Plas'ti.cu.si'ly.ti.ca. N.L. neut. n. plasticus, plastic; N.L. masc. adj. lyticus, dissolving; from Gr. masc. adj. lytikos, dissolving; N.L. fem. adj. *plasticusilytica*, plastic-degrading.
Description of the new taxon and diagnostic traits	Cells are Gram-stain-negative, aerobic, rod shaped (1.0–2.0×0.5–1μm), and motile with a polar flagellum. The strain grows well at pH 7.0 and 28°C with optimal NaCl concentration of 0.5–2%. It can hydrolyze Tweens 20, 40, 60, 80, and aesculin, but does not hydrolyze starch, casein, or cellulose. Strain SCSIO 80796^T could utilize myo-inositol and acetoacetic acid as carbon sources, but was negative for the reduction of nitrates to nitrites or nitrogen, production of indole, H_2S and coagulation and peptonization of milk. Cells are positive for arginine dihydrolase, urease, oxidase and catalase activity, and also alkaline phosphatase, Esterase (C4), Esterase lipase (C8), *β*-galactosidase and *β*-glucuronidase, lipase (C14), leucine arylamidase, valine arylamidase, cystine arylamidase, acid phosphatase, naphtol-AS-BI-phosphohydrolase, α-Galactosidase, *α*-Glucosidase, *β*-Glucosidase, *N*-Acetyl-*β*-glucosaminidase. Its primary respiratory quinone is Q-8, and the major fatty acids are $C_{16:0}$, $C_{16:1}$ *ω7c*/*ω6c* and $C_{18:1}$ *ω7c*/*ω6c*. Strain SCSIO 80796^T contents the phospholipids of phosphatidylethanolamine (PE), phosphatidylglycerol (PG), one

	unidentified phospholipid (PL), and two unidentified lipids (L). The whole genome of SCSIO 80796^{T} consists of 4,456,699 bp and the G+C content is 46.0 %. Strain SCSIO 80796^{T} can produce the novel PET-degrading enzyme *Nm*Cut, which showed well degrading activity at 60°C.
Country of origin	China
Region of origin	the South China Sea
Date of isolation	2022
Source of isolation	mangrove sediment
Sampling date	2022
Latitude	22°25'51"N
Longitude	113°37'48"E
16s rRNA gene accession	PP212059
Genome accession number	CP150481
Genome status	complete
Genome size	4.46Mbp
DNA G+C content (%)	46.0
Number of strains in study	1
Designation of the type strain	SCSIO 80796^{T}
Strain Collection Numbers	MCCC 1K08369^{T} = KCTC 92826^{T}

Table S6 Sequences of 17 well-known plastic-degrading enzymes used to construct HMM profiles.

Enzyme	Sequence
LCC	SNPYQRGPNPTRSALTADGPFSVATYTVSRLSVSGFGGGVIYYPTGTS<br>LTFGGIAMSPGYTADASSLAWLGRRLASHGFVVLVINTNSRFDYPDSR<br>ASQLSAALNYLRTSSPSAVRARLDANRLAVAGHSMGGGGTLRIAEQN<br>PSLKAAVPLTPWHTDKTFNTSVPVLIVGAEADTVAPVSQHAIPFYQNL<br>PSTTPKVYVELDNASHFAPNSNNAAISVYTISWMKLWVDNDTRYRQF<br>LCNVNDPALSDFRTNNRHCQ
TfCut	ANPYERGPNPTDALLEASSGPFSVSEENVSRLSASGFGGGTIYYPRENN<br>TYGAVAISPGYTGTEASIAWLGERIASHGFVVITIDTITTLDQPDSRAEQ<br>LNAALNHMINRASSTVRSRIDSSRLAVMGHSMGGGGTLRLASQRPDL<br>KAAIPLTPWHLNKNWSSVTVPTLIIGADLDTIAPVATHAKPFYNSLPSS<br>ISKAYLELDGATHFAPNIPNKIIGKYSVAWLKRFVDNDTRYTQFLCPG<br>PRDGLFGEVEEYRSTCPF
PET2	ANPPGGDPDPGCQTDCNYQRGPDPTDAYLEAASGPYTVSTIRVSSLVP<br>GFGGGTIHYPTNAGGGKMAGIVVIPGYLSFESSIEWWGPRLASHGFVV<br>MTIDTNTIYDQPSQRRDQIEAALQYLVNQSNSSSSPISGMVDSSRLAAV<br>GWSMGGGGTLQLAADGGIKAAIALAPWNSSINDFNRIQVPTLIFACQL<br>DAIAPVALHASPFYNRIPNTTPKAFFEMTGGDHWCANGGNIYSALLG<br>KYGVSWMKLHLDQDTRYAPFLCGPNHAAQTLISEYRGNCPY

Enzyme	Sequence
TfCut	ANPYERGPNPTDALLEARSGPFSVSEENVSRLGASGFGGGTIYYPREN<br>NTYGAVAISPGYTGTQASVAWLGKRIASHGFVVITIDTITTLDQPDSRA<br>RQLNAALDYMINDASSAVRSRIDSSRLAVMGHSMGGGGSLRLASQRP<br>DLKAAIPLTPWHLNKNWSSVRVPTLIIGADLDTIAPVLTHARPFYNSLP<br>TSISKAYLELDGATHFAPNIPNKIIGKYSVAWLKRFVDNDTRYTQFLCP<br>GPRDGLFGEVEEYRSTCPF
Est119	ANPYERGPNPTESMLEARSGPFSVSEERASRFGADGFGGGTIYYPREN<br>NTYGAIAISPGYTGTQSSIAWLGERIASHGFVVIAIDTNTTLDQPDSRA<br>RQLNAALDYMLTDASSAVRNRIDASRLAVMGHSMGGGGTLRLASQR<br>PDLKAAIPLTPWHLNKSWRDITVPTLIIGAEYDTIASVTLHSKPFYNSIP<br>SPTDKAYLELDGASHFAPNITNKTIGMYSVAWLKRFVDEDTRYTQFL<br>CPGPRTGLLSDVEEYRSTCPF
TcCut	ANPYQRGPDPTEASLRAPRGPFAVSEQSVSRLSVSGFGGGTIYYPTDTS<br>QGTFGAIAISPGFTASWSSLAWLGPRLASHGFVVIGIETNTRYDQPDSR<br>GRQLLAALDYLTQRSSVRDRVDASRLAVAGHSMGGGGTLEAARRRP<br>SLKAAIPIAPWNLDKTWPEVTTPTLIIGGENDSIAPVATHAIPFYNSLTN<br>ATEKAYLELNGASHFFPQTPNDTMAKFMIAWMKRFIDDDTRYDQFLC<br>PPPRPSGDISEYRDTCPHT
SvCut	GPQDNPYERGPDPTEDSIEAIRGPFSVATERVSSFASGFGGGTIYYPRET<br>DEGTFGAVAVAPGFTASQGSMSWYGERVASQGFIVFTIDTNTRLDQP<br>GQRGRQLLAALDYLVERSDRKVRERLDPNRLAVMGHSMGGGGSLEA<br>TVMRPSLKASIPLTPWNLDKTWGQVQVPTFIIGAELDTIAPVSTHAKPF<br>YESLPSSLPKAYMELDGATHFAPNIPNTTIAKYVISWLKRFVDEDTRY<br>SQFLCPNPTDRAIEEYRSTCPY
MtCut	SNPYERGPAPTESSVTAVRGYFDTDTDTVSSLVSGFGGGTIYYPTDTSE<br>GTFGGVVIAPGYTASQSSMAWMGHRIASQGFVVFTIDTITRYDQPDSR<br>GRQIEAALDYLVEDSDVADRVDGNRLAVMGHSGGGGTLAAAENRPE<br>LRAAIPLTPWHLQKNWSDVEVPTMIIGAENDTVASVRTHSIPFYESLD<br>EDLERAYLELDGASHFAPNISNTVIAKYSISWLKRFVDEDERYEQFLCP<br>PPDTGLFSDFSDYRDSCPHTT
PHL7	ANPYERGPDPTESSIEAVRGPFAVAQTTVSRLQADGFGGGTIYYPTDT<br>SQGTFGAVAISPGFTAGQESIAWLGPRIASQGFVVITIDTITRLDQPDSR<br>GRQLQAALDHLRTNSVVRNRIDPNRMAVMGHSMGGGGALSAAANN<br>TSLEAAIPLQGWHTRKNWSSVRTPTLVVGAQLDTIAPVSSHSEAFYNS<br>LPSDLDKAYMELRGASHLVSNTPDTTTAKYSIAWLKRFVDDDLRYEQ<br>FLCPAPDDFAISEYRSTCPF

Enzyme	Sequence
611	AEPADVHGPDPTEESITAPRGPFEVDEESVSRLSVSGFGGGTIYYPTDT<br>TDGLFSAVSISPGFTGTQETMAWYGPRLASQGFVVFTIDTITTTDQPDS<br>RARQLQASLDYLVNDSDVKDIIDPARLGVMGHSMGGGGSLKAALDN<br>PALKAAIPLTPWHTTKDFSGVQTPTLIIGAQNDTVAPVSQHAKPFYESL<br>PDDPGKAYLELAGASHLAPNTDNTTIAKFSIAWLKRFLDDDTRYDQF<br>LCPPPENDDSISDYQSTCPY
RgPETase	KLNRLFQVACLAATLVTATAASAVQIGPAPTKASLEASRGPFTVATTR<br>LSANGHGGGTIYYPTNAGAKVGVIAIVPGYLSYQSSIEWWGPRLASH<br>GFAVVTIDTLTIYDQPSSRSSQQLRALDQVVALGSKSTSPLYNKVDGS<br>RTGVMGWSMGGGGSLISAQNRPSIKAAAPQAPWNTTSNFSSLTVPTLI<br>FACQADVVAPILSHAVPFYNSMSRNPKQYLERTAGDHFCFNNANPTV<br>GLKGVAWMKRFIDGDTRYTSFACSNPNALGFSSFRTERCS
CaPETase	ADNPYQRGPDPTNASIEAATGPFAVGTQPIVGASGFGGGQIYYPTDTS<br>QTYGAVVIVPGFISVWAQLNWLGPRLASQGFVVIGIETSVITDLPDPRG<br>DQALAALDWATTRSPVASRIDRTRLAAAGWSMGGGGLRRAALQRPS<br>LKAIVGMAPWNGERNWSAVTVPTLFFGGSSDAVASPNDHAKPFYNSI<br>TRAEKDYIELRNADHFFPTSANTTMAKYFISWLKRWVDNDTRYTQFL<br>CPGPSTGLFAPVSASMNTCPF
BhrPETase	SNPYQRGPNPTRSALTTDGPFSVATYSVSRLSVSGFGGGVIYYPTGTTL<br>TFGGIAMSPGYTADASSLAWLGRRLASHGFVVIVINTNSRLDFPDSRA<br>SQLSAALNYLRTSSPSAVRARLDANRLAVAGHSMGGGATLRISEQIPT<br>LKAGVPLTPWHTDKTFNTPVPQLIVGAEADTVAPVSQHAIPFYQNLPS<br>TTPKVYVELDNATHFAPNSPNAAISVYTISWMKLWVDNDTRYRQFLC<br>NVNDPALSDFRSNNRHCQL
IsPETase	NFPRASRLMQAAVLGGLMAVSAAATAQTNPYARGPNPTAASLEASA<br>GPFTVRSFTVSRPSGYGAGTVYYPTNAGGTVGAIAIVPGYTARQSSIK<br>WWGPRLASHGFVVITIDTNSTLDQPSSRSSQQMAALRQVASLNGTSSS<br>PIYGKVDTARMGVMGWSMGGGGSLISAANNPSLKAAAPQAPWDSST<br>NFSSVTVPTLIFACENDSIAPVNSSALPIYDSMSRNAKQFLEINGGSHSC<br>ANSGNSNQALIGKKGVAWMKRFMDNDTRYSTFACENPNSTRVSDFR<br>TANCS
KubuP	ADQVGQAPTAANITGDGSFATASAPITNQTGFGGGTVYYPTAAGTYP<br>VVAVVPGFVSTWSQISWLGPRVASWGFVVVGADTTSGFDSPSQRADE<br>LLAALNWAVNSAPAAVRGKVDGTRRGVAGWSMGGGGTLEALAKD<br>TTGTVKAGVPLAPWDIGQDFSKVTKPVFIVGAQNDTIAPPAQHAVPFY<br>NAAAGPKSYLELAGADHFFPTTANPTVSRAMVSWLKRFVSSDDRFTP<br>FTCGFAGAAVSAFRSTAC

Enzyme	Sequence
MipaP	APPASATERGLAPTAANITGDGSYGVVSATITGASGFGGGVVYYPNA<br>TERFPVVAISPGYTERWSSFAWLGRRLASWGFVVVGIETNSLFDQPNS<br>RGTQLLRALDWASSSAPAAVRDRVDATRQGVSGHSMGGGGTLSAM<br>DQRPSVRAGVPLAPWHTTTSWPRVTNPVMILGGQNDGIAPVSSHAIP<br>MYTGVASGEKAYVELAGAGHNFPNSANPIVSRAAVSWFKRFLDDDT<br>RFAPFACDFGGASISQFRSTCPV
FsC	MKFFALTTLLAATASALPTSNPAQELEARQLGRTTRDDLINGNSASCR<br>DVIFIYARGSTETGNLGTLGPSIASNLESAFGKDGVWIQGVGGAYRAT<br>LGDNALPRGTSSAAIREMLGLFQQANTKCPDATLIAGGYSQGAALAA<br>ASIEDLDSAIRDKIAGTVLFGYTKNLQNRGRIPNYPADRTKVFCNTGD<br>LVCTGSLIVAAPHLAYGPDARGPAPEFLIEKVRAVRGSA

Table S7 List of candidate plastic-degrading enzymes identified in strain SCSIO 80796^{T} using HMMER. The sequences were further annotated using the Swiss-Prot database and the CLEAN platform.

	full sequence			best 1 domain					
**target name**	**E-value**	**score**	**bias**	**best domain E-value**	**best domain score**	**bias**	**Swissport-product**	**CLEAN**	**Sequence**
SCSIO80796_2283	5.00E-06	24.1	0.3	3.00E-05	21.6	0.3	Dipeptidyl aminopeptidase BIII	EC 3.4.19.1 - acylaminoacyl-peptidase	MRNVFLLSTLLTCFSHVSLAATPSLVTALDDFAQLPAIQHPALSPDGHYLATQCSNGRRFEICIADLTDKQPTIHFGTGDEAQIKHVSWGSNKHVLVYIQQTRDVALRFQTIEVEYDRIYAFNVEQQTGVVLMNGIGGSIRNLTHVVALLPDQESVLMQILLADDNDKLDYRATIYQVDLNDGRSERIKQGALSVRNFVYDAEGKLLAETKLLERHNSFTLDSYLPEKHTIYKQDTAIVPFSVMGMVDSGKIAVQFDDQRGAMSLSLADGQLDPLTFQGEIVGQTGSAITDDYRNTLLGFEFWDGVLPDQMFTHGRFAGLKKALAQAMPDKKVLIVSWTRDLKTFLLKATEAGKPDDFYTFSLNTGMMDALGTSSTLPAEHVFSQVMPIEYQANDGLKIHGFVTLPPGKTVKQGPFPMILMPHGGPESHDDASYDWMAQYYASLGYAVLQPNFRGSSGYGVEFRNKGFGEYGGKMVTDVIDGYHNMVKKGIAAKNGFCAIGWSYGGYSALQMAVKSADLIKCAISVNGVTDPVHQKKYRSQVGSQQWYYYDQYLGLENNQGKIRSYTPLENVEQIKAPVLLLHGNQDSTVPFIQAEKFRDAMQTYNKVPFRFVEVDAQDHYFQDVAARKTVLTETTEWLKMYFPLKTTQSKSEKPE
SCSIO80796_2563	8.50E-06	23.4	0	1.20E-05	22.8	0	Leaf-branch compost cutinase	EC 3.1.1.1 - carboxylesterase	MAKHSQFPQWQVAQQELVINILSATISKRWTIRRVIRIGFILWALIAMLWLANSVRTQGVDKSTLQSNSLISVQNNATSLAFLPSSPNSHAGLIFICGSGVAAEAYAPLLRPIAEDGTPVFIVKLPYRFAPRDTDKNAAIGRVDKVMVSHPEVKYWVVAGHSLGGALTAKLAMETPELLSAIILIATTHPKDYDLSRLDIPVIKIYASNDGIAPLERVLANKDRLPSHTRWLELKGGNHSQFGRYGHQLLDGTATISREEQEAFTRSAIRRVLADIKKG
SCSIO80796_0503	0.00076	17	0	0.0015	16	0	Dipeptidyl aminopeptidase 4	EC 3.4.14.5 - dipeptidyl-peptidase IV	MSMPRPVLGLLLVLSMAGCSSTINTHSNSMTNEMSATPNKLTLENLYKNKTYATKKLGSVRWMQDGVSYTAVEKSADIEDAQDIVRYQAGNDQRQILVSAKTLIPEGQDKPLEIDDYQWNSDQSQLMLYTNSQKVWRSKSRGDYWLLDINSGKLTQLGEGKQKAEEATMMFAKFSPDDKSVAYVRDNNIYVQSLTDYSIQQLTQDGKGHIINGIFDWVYEEEFSIADGFRWSPDSQRIAYWQLDTSAVKDFTMINNTDALYPTLTVFPYPKAGEKNATVRIGIAALASGKTQWAKIPADTDDFYIPRMNWSGNSDEILVQKVNRLQNHNDHYLVDVKTGKARNIFVDADKAFIERYYDVKW

									LNDGKQFLKISERDGWRHVYLVSRDGKQIKDITPGDFDITEIVHVDEKQGDIYFTSGKDNVIEQYLYRANLDGSGDIERVTPEKYKGSNGYQISPDGKLAVQTYSSFRTPTQYHIISLPEHKNIRTLIDNHQVTEALSGIAMSDMEFFRVPSYDGTMLDGMIMRPADFDPSKKYPILFYVYGEPAGQTVKNSWGRSGPLWFEYLTEQGFIVASIDNRGTRSPRGREWRKQIYRHIGTLAAKDQATALDEMAKRWSYIDTSRVGIWGHSGGGSMTLNMLFRYPEKYKVGIAGAPVPDIRLYDTIYQERYMGLPDTFTTETGREAYDQASPITFAENLQGKLLLIHGTGDDNVHYQGSERLINKLVEYNKQFEFMSYPNRSHSLREGKGTSLHQMTMMANFFKTNLLESGH
SCSIO80796_2105	0.001	16.6	0	0.0037	17	0	Esterase YbfF	EC 3.1.2.1 - acetyl-CoA hydrolase	MKLLNFQEKGAGEAIVLLHGLLGSLDNLGMVTRGIESSLRVIAVDLPDHGQSYFSEQFSYAEYANAVLQLLDHLGIDRCHLVGHSMGGKVAMQLALNHPQRVTKLVVADIAPVAYPAHHTAIFAGLNNVDLATISNRNDADNALAKHISEAGVRQFLLKSLQQTEQGWQWRFNLPLLQRDYALISAGIDSSQTFEQPTLFIKGTESDYIQAEHRESIQRLFPNAKAQLMQGCGHWLHAEKPQQFNRILLNFIHP
SCSIO80796_1158	0.0014	16.6	0	0.0014	17.1	0	Dipeptidyl aminopeptidase 4	EC 3.1.1.72 - acetylxylan esterase	MLKQRYRNILLLSILLCIGHFSWAEPSPRFEEGQFKQLDYRILYPADFDPAKQYPLVLVLHGAGERGDDNKAQLVHGSQLFLQPQVREKFPAIVIFPQAPKSDYWANVDVDRSGPRPKFSFKDGGKPTTSMALVMELMDNFSSQSYVDNSRIYVGGLSMGGMGTFEILSRKPDIFAAAFAICGGGDPAIVSRYRNGLPIWIFHGEKDDIVSPRYSRQMADAISKQGGKVKLTLYPEANHNSWDSAFAEPQLLPWLFAQRLTR
SCSIO80796_3201	0.0016	15.8	0	0.0025	15.3	0	Putative hydrolase fragment YghX	EC 3.1.1.45 - carboxymethylenebutenolidase	MPKHPKLPEQAFTLYDQFAHGRINRRAFLSGLGKLSVGALTTSIMLDALMPNYALAEQVSFNDPDIIASYQEFSSPDGYGTGKGYRVEPSKLDKPAPLVLVIHENRGLNPYIKDVARRFAKQGFIAFAPDGLYPVGGYPGNDEQGKKMQASMDKDKLANDFFAAAKSLKGDGKGNGKLGVIGFCYGGGMVNKIVTEAPDLADAAVPFYGAAPDLAKVK
SCSIO80796_0871	0.0017	15.8	0	0.0025	15.3	0	Macro domain-containing protein	EC3.1.1.106 - O-acetyl-ADP-ribose deacetylase	MSAKLTLLQGDICQLAVDAIVNAANSSLLGGGGVDGAIHRAAGPDLIAECKTLGGCETGQAKLTQGYLLPAKYVIHTVGPVYRDGHQNEARLLASCYQNSIALAEQYQLRSLAFPAISCGVYGYPLDEACRIAVNAITESCACYPNIRKVILCAYDDKVHQAWLSALQSSQHH
SCSIO80796_3782	0.0038	14.7	0	0.0047	14.5	0	Carboxylesterase 2	EC 3.1.1.1 - carboxylesterase	MSDNLLPYVEVNPDKPATAVVIWLHGLGDSGNGFAPIVPELNLPEELAIRFVFPHAPIRAITINNGYQMRAWYDIKSMELDKRADAAGVRESAELVKTLIEREIAQGIEASRIVLAGFSQGGVIALHLATRFEQKLAGVMALSTYMSEPDTLEQQAHPAN
SCSIO8079	0.011	13.2	0.3	0.069	10	0.3	Acyl-coenzyme A	EC 3.1.1.1 - carboxylesterase	MQTDTFTTVVIENDTLAGHLYIPANSEGVPVVIVLGGSGGGLSNVKGELLAQNGIAVLSLAYFRYKHLPETLDGIAVEYVINAINYLHNMPVFQTSKIGIWGASRGSELAFLAATHDSRIKSLVVTTPSKVAWHGATTPVAWTYNNLAVASLTFDK

6_0232							thioesterase 2		ASDTAVIDKSSMALENPVNVARAQFRFEKINGPILLVSAERDQIWPSFQMARDIEKYLALHQFKHKVIHQSYPTGHTFSQHYWPAISASIVEHFNRSLR
SCSIO80796_2388	0.011	13	0	0.026	12	0	Proline iminopeptidase	EC 3.4.11.5 - prolyl aminopeptidase	MTKIIWLLLATFCLAGCAQNGSHYLDNAGRSDILTGGVQMIPLDTPKGPFHVWTKRTGNNPTIKVLLLHGGPGFNHEYLEAFDSFFPAANIEYYYYDQLGSYYSDQPEDPDLWDLARFVEEVEQVRQALKLDEDNFYLYGHSWGGLLAIEYALKYQQHLKGLIIANMGPSIPAYNDYANKVIMPAMDPQVLAQIKAYEAANDYDDPNYMALLFEHHYSLHILRMPPDDWPEPVQRSFRHMNPAVYVPMQGPSELGASGKLVDWDRTADLANIGVPTLAIGARYDTMEPAQMELIADKVQQGRYLYCPNGSHMALYDDQEVFFQGLIKFIKDVDKGNFK
SCSIO80796_1097	0.012	13	0	0.018	12.5	0	S-formylglutathione hydrolase YeiG	EC 3.1.2.12 - S-formylglutathione hydrolase	MSQLEQTSSSKLFGGWQNQYQHQSSCLNCTMRFSVYLPPQAESGQRLPVLYWLSGLTCSDENFVTKAGAQCVAAELGIILVIPDTSPRGDEVPDDEAYDLGKGAGFYVNATQAPWDQHYHMYDYIVNELPALITAHFNVRDKAAIAGHSMGGHGALVIAL
SCSIO80796_1452	0.035	11.6	0	0.05	11	0	Probable carboxylic ester hydrolase LipM	EC 3.1.1.1 - carboxylesterase	MKTTGMSQRFLRIMAVLLLTFQTHAFEVDSSYTLASSFQKYHQQYPQLAIADLRLADRVRVELDREYANIEGRTLHLDLFVPHNRPPKATVILIHGGGWRSGNKSHFFPLAGALANRGYLTVTVEYRLSVEAPYPAGLIDINRAIVWLKNHAENYQIDVSKMALMGGSSGGHMAALLANTADLQLYRPPEISGDTQVQAVIDMDGILDLTSDLGLKYEDKNGRKDTAMGLWLEGNYASQTARWQQVSPAYYITGNSPPMLFISSGQARFAEGHDKV
SCSIO80796_1339	0.041	11.3	0	2.3	5.6	0	Putative 2-succinyl-6-hydroxy-2,4-cyclohexadiene-1-carboxylate	EC 3.1.1.1 - carboxylesterase	MRPNNQTVLAGVKISGAGRPLVLLHSSMSSGKQWTKLVQQLKDNHQIINIDLLGYGDAPQPDRNTVFSLQYETRRIESILAELGVEQFDLVGHSYGGATALKFTYENPSRVNRLVVFEPVAFHLLAADNPARQDVVQLGNSMAEMSDFEAAESFLDYWNGQGYFASIPDFVRQPLLNRVYKGVLDFTALLGESYQLSDYANIVQPTLLLSGEKTRRSAQAVVHELSQHLPNSHMQVTPGGHMAPISHADTVNQYIVDFLD

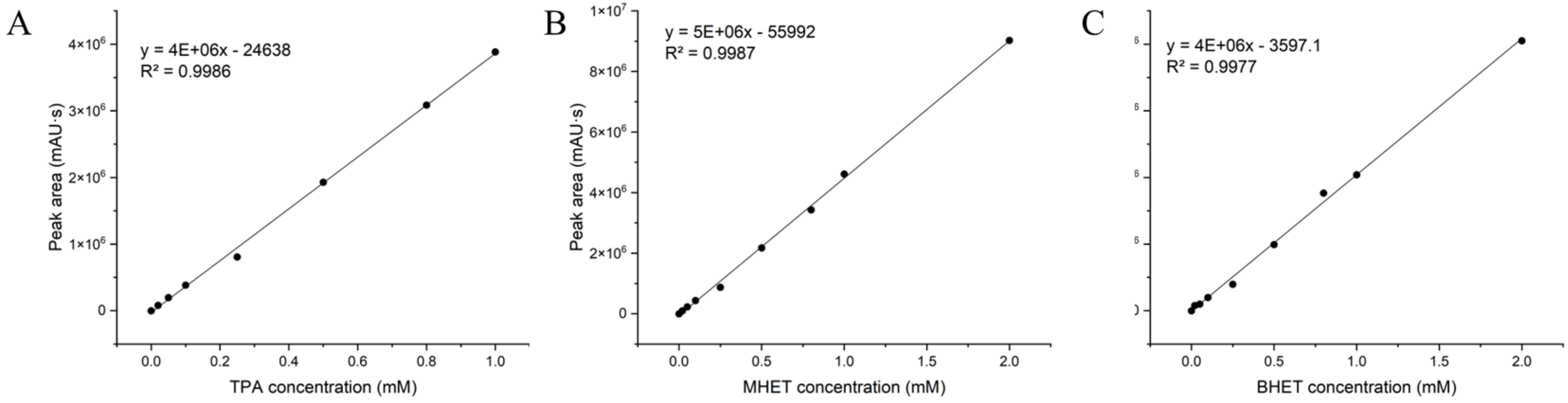

Figure S6. Standard curves for PET degradation products. (A) Calibration curve of (TPA). (B) Calibration curve of mono(2-hydroxyethyl) terephthalate (MHET). (C) Calibration curve of bis(2-hydroxyethyl) terephthalate (BHET).

Peak areas were determined by HPLC at 240 nm and plotted against known concentrations to enable quantification of degradation products in enzymatic assays.

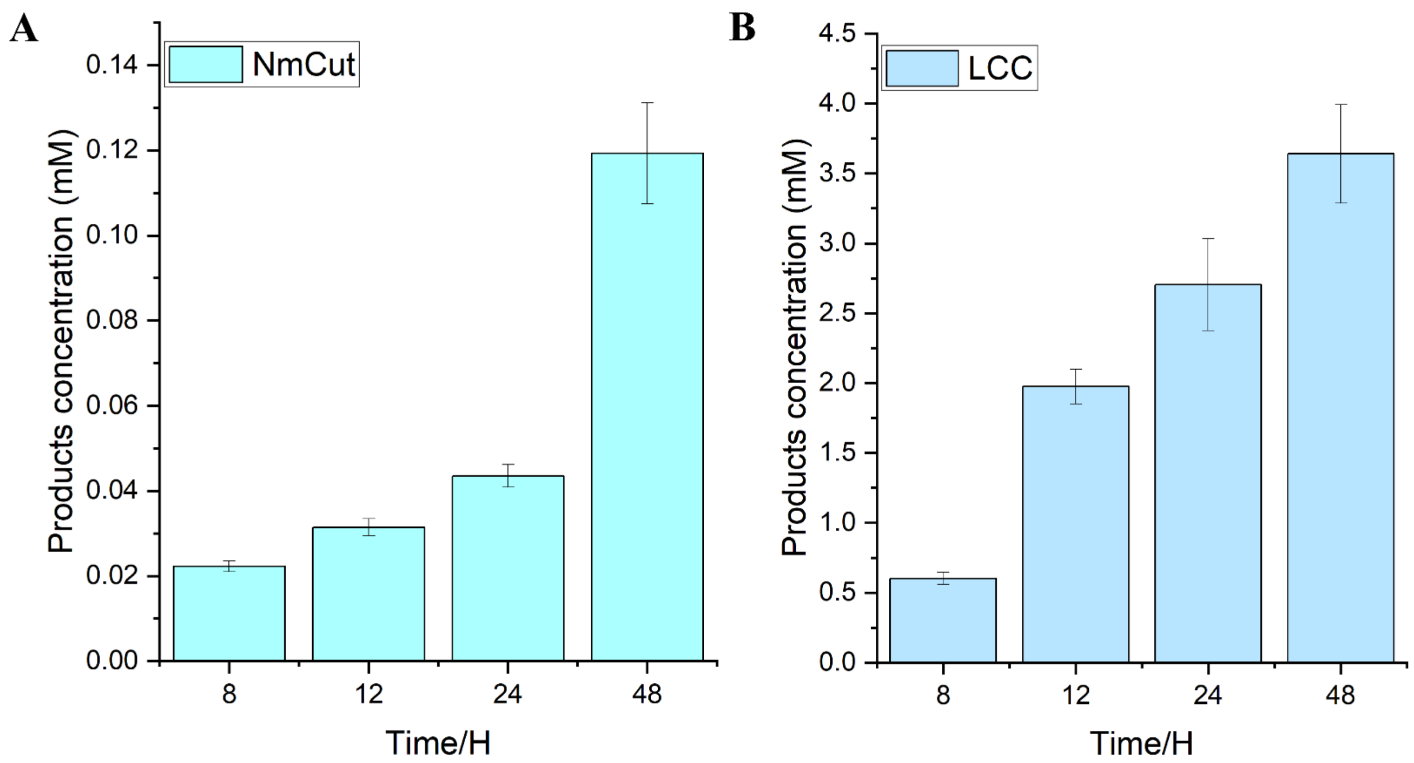

Figure S7. Time-course quantification of degradation products (TPA, MHET, BHET) released from 11.3% crystallinity PET powder incubated in pH 8.0 potassium phosphate buffer at 60 °C over 48 hours. (A) NmCut; (B) LCC. Data are shown as mean ± SD (n = 3).

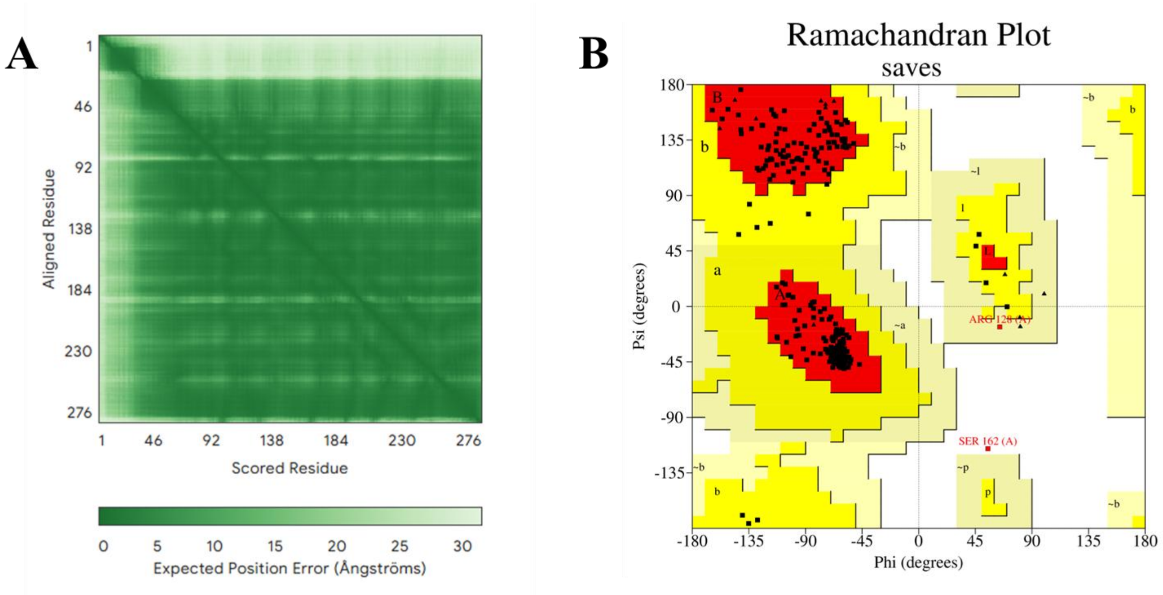

Figure S8. Structural validation of the predicted *Nm*Cut model. (A) AlphaFold3 Predicted Aligned Error (PAE) plot. The color scale indicates the expected positional error (in Ångströms) between residue pairs, with darker green representing lower error and higher confidence. (B) Ramachandran plot generated by PROCHECK. Residues are plotted according to their φ and ψ backbone dihedral angles. Regions are colored as follows: red for most favored, yellow for additionally allowed, and white for disallowed regions.
